# Does an antimicrobial stewardship program for Carbapenem use reduce Costs? An observation in Tehran, Iran

**DOI:** 10.22088/cjim.11.3.329

**Published:** 2020-05

**Authors:** Mahboubeh Hajiabdolbaghi, Jalil Makarem, Mohammadreza Salehi, Seyed Ali Dehghan Manshadi, Esmaeil Mohammadnejad, Hossein Mazaherpoor, Arash Seifi

**Affiliations:** 1 Department of Infectious Diseases, Faculty of Medicine, Tehran University of Medical Sciences, Tehran, Iran; 2 Department of Anesthesiology and Critical Care, Tehran University of Medical Sciences, Tehran, Iran; 3 Department of Nursing and Midwifery, Tehran University of Medical Sciences, Tehran, Iran

**Keywords:** Anti-infective agents, Antimicrobial stewardship, Carbapenem, Drug resistance, Iran

## Abstract

**Background:**

Inappropriate administering of antimicrobials has led to increased antibiotic resistance as well as burden of infectious diseases. Antibiotic stewardship programs (ASPs) help prevent resistance through improved utilization of antimicrobial agents while potentially decrease costs of treatment.

**Methods:**

We reviewed 186 infectious disease (ID) consultations from two internal disease wards in a tertiary center where ID specialists were asked to confirm carbapenem use in patients within 48 hours of initiation. The records were reviewed in terms of age, gender, and final decision about carbapenem use. The crude mortality rates during the 5-month period of the study (May to September 2016) as well as hospital spendings were compared with the same time of the year before the implementation of the ASP.

**Results:**

Of the 186 consultations conducted by the ID specialists, 28 (15%) consultations led to antibiotic change, 46 (25%) led to discontinuation, while 112 (60%) carbapenems were continued. An estimate of 14,000 € was saved based on the annual hospital costs during the 5-month period of the study. Although antimicrobial resistance patterns could not be evaluated, the crude mortality rate in the two IM wards was calculated to be 2.6% with no significant change compared to previous year (CMR: 2.9%).

**Conclusion:**

Based on findings of the present study, ASPs for carbapenems (as wide-spectrum agents) can lower costs with no increased mortality rates in a tertiary center located in a middle-income country.

The discovery of antibiotics in the early 20th century has transformed healthcare provision with dramatic decrease in morbidity and mortality caused by infectious diseases. However, the increasing antimicrobial resistance to available medications besides the slow development of newer agents are the major threats to those achievements (1). Besides, it seems most physicians needed additional training to prescribe antibiotics properly (2). Several organizations including the Infectious Disease Society of America (IDSA), the Society for Healthcare Epidemiology of America (SHEA), and the American Society of Health System Pharmacists (ASHP), have suggested antimicrobial stewardship programs as having a pivotal role in health care environment in the presence of the peril of antimicrobial resistance (3, 4). The IDSA describes antimicrobial stewardship as a “rational, systematic approach to the use of antimicrobial agents to achieve optimal outcomes” (3). The proposed benefits include improved patient outcomes, reduced adverse events including *clostridium difficile* infection (CDI), improvement in rates of antibiotic susceptibility to targeted antibiotics, and improvement of resource consumption across the continuity of care (5). 

IDSA and SHEA strongly believe that antibiotic stewardship programs (ASPs) are best directed by infectious disease physicians with additional trainings (6, 7). ASPs may include various activities such as replacement of cost-effective antimicrobials of the same class, switching from intravenous to oral drugs with high bioavailability, as well as pharmacokinetic consultations that influence the choice of antibiotics (8). For successful implementation of ASPs, at least one trained infectious disease (ID) specialist should be responsible for planning and functioning of the system (6, 7). Rapidly increasing rates of antibiotic resistance and specifically carbapenem resistance in organisms such as *Acinetobacter baumannii* and *Klebsiella pneumoniae* have been well-described (9, 10). The reasonable use of carbapenems is a particular concern because they are often used as the last line of defense against gram negative bacilli, and these organisms had been increasingly reported in healthcare associated infections (11). On the other side, another concern about the broad spectrum antibiotics such as carbapenem is the unnecessary use which has led to additional costs as well as significant morbidity and mortality. Considering the global issue of carbapenem resistant organisms and the consequences in healthcare provision in infection control, we established a study to evaluate the efficacy of a local antimicrobial stewardship program on the optimal treatment of patients with carbapenems. Our aim was to evaluate the effects of the new ASP in a tertiary center located in a middle income country; the focus was mainly on costs of treatment after initiation of the program and we tried to assess crude mortality rates before and after initiation since concerns regarding discontinuation of wide-spectrum antibiotics may be a challenge on the way of successful implementation of such programs.

## Methods

**Study design & Setting:** In a prospective observational study, we reviewed all infectious disease consultations for the continuation of carbapenem antibiotics from two internal medicine (IM) wards in a major referral and tertiary center located in the capital city of Tehran. The duration of the study was five months, starting from May to September 2016. The program had just been in a place and the effects were compared for similar parameters observed in the same period of the previous year before the implementation of the program. 

**Antimicrobial stewardship program (ASP):** Since April 2016, a stewardship program for carbapenem had been devised and approved by the institution’s board with three major goals: control of antimicrobial prescription; prevention of resistance to antimicrobials; and, improvement of patient safety. This program obliged physicians to send stewardship consultation to responsible ID specialists whenever a carbapenem was considered for patients. Carbapenems could only be prescribed if an ID specialist approved it. Structured forms had been designed to gather data on primary diagnosis, culture results, isolated organisms, as well as the name, dosage, cause of prescription, and probable duration of treatment of the prescribed antimicrobial agents. The consultation form had to be completed in 48 hours.

**Inclusion & exclusion criteria:** For the purposes of the current study, inclusion criteria were as follows: admission to the IM wards during the investigation period, initiation of the carbapenem antibiotic by the IM service, completion of an ID consultation from that sent by the IM service. Patients and their records were excluded if the patient had been transferred to other hospital wards or if the patient had died before the ID consultation completed. 

**ASP consultation forms:** Patients participating in the study were evaluated in terms of age, gender, time interval between initiation of a carbapenem antibiotic and ID consultation, and the final decision made after the consultation which would lead to discontinuation, modification, or continuation of carbapenems. The findings were documented through standard forms designed for the stewardship program. 

**Outcome measures and data analysis:** Data extracted from the forms were entered into the *statistical package for social sciences* (SPSS) version 16.0. Descriptive demographic data were presented in terms of frequency and percentage. As indicated earlier, the major goal of the study was to provide an early assessment of a new stewardship program in a tertiary center. To meet that purpose, we included crude mortality rates before and after initiation of the program and compared the two rates utilizing student’s t-test. We also compared the costs of treatment in the two wards before and after initiation of the program. 

**Ethical Considerations:** The study protocol was approved by the *Institution’s Review Boards (IRB)*. The consultation forms were reviewed by an explorer and the data were entered from secondary sheets for analysis. Since the data utilized in the study were extracted from standard ASP forms and there was no direct interaction with the patients and no need for any procedures, informed consents were not recognized essentially by the IRB. 

## Results

**Fig 1:**
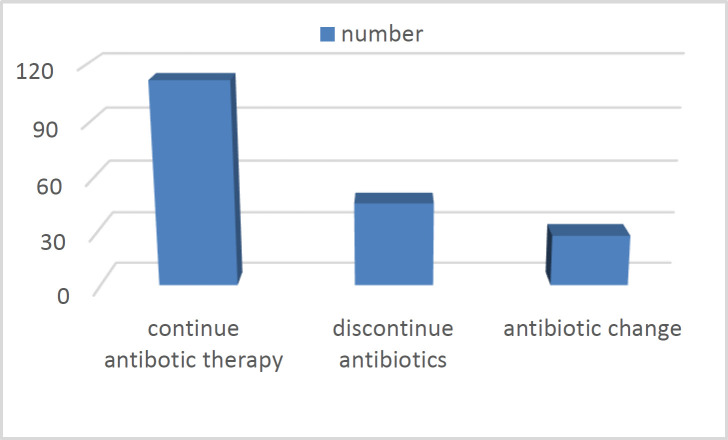
Final decisions of stewardship consultations in two internal medicine wards

In total, 186 patients admitted to IM wards from May to September 2016 had required stewardship consultation based on specialist visits. From them, all 186 forms were reviewed by our research team. In total, 109 patients were men (59%) and 77 were women (41%). The age distribution of participants in the study showed that most patients were between the ages of 61 to 80 (36%). Out of the total 186 consultations provided by the ID specialists for patients who were treated by carbapenem, 28 (15%) consultations led to an antibiotic change, 46 (25%) consultations led to discontinuation of carbapenems, and there was no change in 112 (60%) consultations (figure 1). Also of note was the delay in sending consultations after the initiation of carbapenems; almost 83% were requested after 24 hours from treatment.

As explained in the methods section, the summation of costs of hospital care and treatment were compared in the 5-month period of the study and compared with the same time of the previous year. Cessation of carbapenems in the current study led to a 14,000 Euros reduction in costs. In this research, the implementation of antibiotic stewardship programs led to 25% drop in the unnecessary use of carbapenems. The crude mortality rate was measured to be 2.6% in the patients evaluated during 5 months; this figure was lower than the 2.9% rate reported in the previous year, although the change was not statistically significant.

## Discussion

The findings of the present study highlight the effects of stewardship programs in the reduction of hospital costs along with consistent mortality rates. The study has been an early assessment after the initiation of an ASP while the findings approved the general framework. The findings are in line with those of the developed countries (1). 

Given that proper administration of antibiotics, especially broad-spectrum antibiotics such as carbapenems, are of great importance (9). This study was designed to evaluate the use of carbapenems in the general IM wards of a major referral academic center in Tehran. During the assessment period, one of the problems with the implementation of antibiotic stewardship in these sections was the delay in consultation; according to the results, 83% of the consultations were requested by the IM service after 24 hours of antibiotic initiation. 

When evaluating the final decisions of the ID specialists after consultations, we observed that another issue was the inappropriate administration of carbapenems; in 25%, carbapenems were discontinued while in 15% of cases, they were changed to another antibiotic class. Several reasons are assumed as the causes of improper administration of carbapenems including the unawareness about the range of antibiotic effects, unfamiliarity with alternative antibiotics with similar efficacy, narrower spectrum, easier administration, and fewer side effects, unavailability of an appropriate alternative antibiotic, interest in reducing the frequency of antibiotic administration during the day, and unawareness of the importance of increasing resistance to carbapenems.

Implementation of ASPs has faced similar challenges in different settings such as lack of cooperation on the side of the responsible physicians with ID specialists to change or stop antibiotics already prescribed, unavailability of appropriate alternative antibiotics, and inattention to the threatening possibility of resistance to broad-spectrum antibiotics (1, 3, 4). This study was conducted to determine the status of prescribing carbapenems and antibiotic stewardship on a small scale for the first time in our tertiary center. 

In future studies, antibiotic stewardship for other antibiotics on a wider scale may also be considered, as well as evaluating the potential problems with antibiotic stewardship and making possible solutions for these problems.

In conclusion increasing resistance to antibiotics is a major health concern in society and one of the main causes of this resistance is the inappropriate use of antibiotics. Findings of the present study indicate that the organized use of antibiotics through stewardship programs can save money with no increased mortality.
